# Induction of Macrophage-Like Immunosuppressive Cells from Mouse ES Cells That Contribute to Prolong Allogeneic Graft Survival

**DOI:** 10.1371/journal.pone.0111826

**Published:** 2014-10-30

**Authors:** Hiroya Kudo, Haruka Wada, Hajime Sasaki, Hyuma Tsuji, Ryo Otsuka, Muhammad Baghdadi, Satoshi Kojo, Tatsuya Chikaraishi, Ken-ichiro Seino

**Affiliations:** 1 Institute for Genetic Medicine, Hokkaido University, Sapporo, Japan; 2 Department of Urology St. Marianna University School of Medicine, Miyamae-ku, Kawasaki City, Kanagawa, Japan; Rutgers University -New Jersey Medical School, United States of America

## Abstract

Recent progress in regenerative medicine has enabled the utilization of pluripotent stem cells (PSCs) such as embryonic stem cells (ESCs) as a donor resource for transplantation. However, immune suppression is still needed when the donor-recipient combination is allogeneic. Protection of ESCs-derived grafts from host immune response might be achieved thought the utilization of immunosuppressive cells generated from ESCs. In the present study, we show that a certain fraction of immunosuppressive cells can be generated from ESCs and help to suppress immune response against allogeneic grafts. ESCs-derived suppressor cells (ES-SCs) resembled macrophages in terms of cell surface molecule and gene expressions. Furthermore, gene expression analysis including microarray showed that ES-SCs have M1/M2 hybrid phenotype with high expression of genes correlated to immunosuppression of T cell response. Indeed, ES-SCs were effective to block allogeneic T cell proliferation in a nitric oxide-dependent manner, and prolonged the survival of ESCs-derived embryoid bodies or cardiomyocytes grafts transplanted into mouse kidney capsule. Thus, we consider the potential use of these ESCs-derived macrophage-like immunosuppressive cells as cellular therapies to promote long-term graft survival in future therapies.

## Introduction

Organ transplantation is one of the great advances through the history of medicine. The first successful organ transplantation was reported in 1954 of a case of kidney transplantation between identical twins. After this success, organ transplantation was widely expected to help curing patients with end-stage organ dysfunction. However, the problem of immune rejection between donor and recipient of different backgrounds has emerged as one of the biggest obstacles for this therapy.

Recent progress in the field of regenerative medicine has raised the possibility to create organs or tissues from human pluripotent stem cells (PSCs) such as embryoid stem cells (ESCs), or induced pluripotent stem cells (iPSCs) [1], [2]. These advances gave a rise to a new era of transplantation medicine by utilizing these cells as a renewable source of grafts for replacement therapy. However, allogeneic immune rejection still consist a major problem for the future of transplanted graft. ESCs are derived from the inner cell mass of embryonic blastocyst, and thus tissues differentiated from ESCs should be allogeneic for most recipients. In this context, iPSCs derived from autologous cells are expected to be immune privileged, while iPSCs derived from allogeneic donor cells will probably undergo immune rejection. With this in mind, a bank of iPSCs collected from donors with various human leukocyte antigens (HLA) haplotype has been planned for future therapeutic use [3]. However, even in cases of similar HLA haplotypes, other minor antigens are exist and can also cause allogeneic immune rejection. Therefore, histocompatibility remains a significant barrier to the clinical application of ESCs and iPSCs.

To overcome the problem of allogeneic immune rejection, various excellent immunosuppressants such as calcineurin inhibitor or mycophenolate mofetil have been developed in the field of organ transplantation [4]. Unfortunately, lifelong use of immunosuppressive drugs has been associated with cumulative side effects including increased risks of infection, cancer and diabetes. For this reason, new approaches based on the induction of specific immune tolerance against transplanted organ may help to serve as alternative strategy to prolong the graft survival. Indeed, recent studies have reported the utilization of various regulatory immune cell populations such as FoxP3^+^ regulatory T cells or costimulation blockade-induced anergic cells to promote tolerance during allogeneic transplantation [5], [6], [7], [8]. Additionally, in clinical renal or hepatic transplantation, cell therapy-based methods have been reported to be able to induce immune tolerance [9], [10], [11]. However, little is known about cell therapy-based immune regulation to grafts derived from PSCs.

Myeloid cells are key immune cells that play dual roles in the promotion and regulation of immune response. In the present study, we hypothesize that the differentiation of myeloid cells with immunosuppressive functions from ES cells may help to suppress immune response against allogeneic grafts. We have evaluated this hypothesis, and found that in a myeloid cell differentiation from mouse ESCs, a certain fraction with substantial immunosuppressive capacity could be generated. With a detailed examination of phenotype and function of this unique fraction, we show that ES-derived immunosuppressive cells are effective to block allogeneic T cell proliferation and prolong ESCs-derived graft survival. Together, the utilization of immunosuppressive cells generated from PSCs may provide a safe and effective future solution to challenges surrounding organ transplantation.

## Materials and Methods

### Mice, cells, and culture media

Six-week old female 129X1/SvJJmsSlc (129; H-2^b^) and C3H/HeSlc (C3H; H-2^k^) mice were purchased from Japan SLC, Inc. (Shizuoka, Japan). All animal procedures were approved by the Hokkaido University Animal Care Committee (Approval number: 11-0129). Mouse ESC (E14) cells were kindly provided by Dr. Satoru Senju (Kumamoto University, Japan). E14 cells were maintained in ESCs media (DMEM high glucose supplemented with 20% knockout serum replacement containing recombinant human leukemia inhibitory factor (LIF)) on feeder layers of irradiated mouse embryonic fibroblasts. OP9 cells (obtained from RIKEN, Tsukuba, Japan) were cultured as monolayers in OP9 media (α-MEM supplemented with 20% fetal bovine serum (FBS)). Cardiomyocytes were differentiated from ESCs as described previously [12].

### Differentiation of ES-DCs and ES-SCs

Myeloid differentiation of ESCs was firstly induced with a withdrawal of LIF from the culture. By day 5 of culture, embryoid body (EB)-like round-shaped spheres were formed. The spheres were disrupted with 0.25% trypsin and the resultant single-cell suspensions were re-plated at a density of 5×10^5^ cells per 100-mm untreated dish containing fresh OP9 cells with the addition of granulocyte macrophage colony-stimulating factor (GM-CSF) and macrophage colony-stimulating factor (M-CSF) (both 10 ng/ml; Peprotech, Rocky Hill, NJ). On day 10 of culture, loosely adherent hematopoietic cells were harvested and transferred to bacteriological petri dishes (5×10^5^ cells/100-mm dish) without feeder cells and cultured in the presence of GM-CSF in RPMI-1640 high glucose supplemented with 0.1 mM non-essential amino acids, 1 mM sodium pyruvate, 100 µM beta-mercaptoethanol, 10% FBS, 100 U/ml penicillin and 100 µg/ml of streptomycin (all from Life Technologies, Carlsbad, CA). On day 15 of culture, loosely adherent cells and floating cells cultured in petri dishes were transferred to new untreated dishes and cultured in RPMI-1640/10% FBS supplemented with GM-CSF (5 ng/ml) and Interleukin-4 (IL-4) (10 ng/ml). On day 20 of culture, floating cells were obtained as ES cell-derived dendritic cells (ES-DCs). On day 22, lipopolysaccharide (LPS) (1 µg/ml; Sigma Aldrich, St. Louis, MO) was added and further cultured for 48 hours, and adherent cells were obtained as ES cell-derived suppressor cells (ES-SCs).

### Endocytosis assay

Bone marrow cells were cultured in high glucose RPMI-1640 supplemented with 1% L-Glutamine, 0.1 mM non-essential amino acids, 1 mM sodium pyruvate, 100 µM beta-mercaptoethanol, 10% FBS, 100 U/ml penicillin and 100 µg/ml of streptomycin (all from Life Technologies, Carlsbad, CA) and 10 ng/ml of M-CSF (Peprotech, Rocky Hill, NJ). Adherent cells were obtained as bone marrow-derived macrophages. AlexaFluor488-labeled *Staphylococcus aureus* (*S. aureus*) particles and *Escherichia coli* (*E. coli*) particles (both from Molecular Probes, Eugene, OR) were opsonized for 24 hours in cell culture medium containing 10% FBS. Opsonized particles (2×10^6^ particles per ml) were added to cell culture and incubated for 90 minutes for *S. aureus* particles and for 3 hours for *E. coli* particles. After incubation, cells were detached and analyzed for the increase of cell-associated fluorescence in comparison to untreated control cells by flow cytometer.

### Micro array analysis

Total RNAs of ES-DCs and ES-SCs were labeled with Cy3 and hybridized to a Whole Mouse Genome Microarray (Agilent) according to the manufacturer’s protocol. Arrays were scanned using the Agilent Technologies Microarray Scanner. Data were analyzed using the MultiExperiment viewer (http://www.tm4.org/). Microarray data are available from the Gene Expression Omnibus (GEO, http://www.ncbi.nlm.nih.gov/geo/) with the accession number GSE56302.

### Mixed lymphocyte reaction and measurement of nitric oxide

Splenic T cells were isolated from C3H mice using a pan-T cell isolation kit (MiltenyiBiotec, Belgish-Bladbach, Germany) and used as responder cells. Bone marrow-derived dendritic cells (DCs) [13] from 129X1/SvJJmsSlc mice were used as stimulator cells. Responders (1×10^5^) were co-cultured with 35 Gy-irradiated stimulators (2×10^4^) in 96-well round-bottomed culture plates. For some experiments, 35 Gy-irradiated ES-DCs or ES-SCs were added to the culture. On day 4 of culture, T cell proliferation was assessed by a carboxyfluorescein diacetate succinimidyl ester (CFSE, DOJINDO, Kumamoto, Japan) or [^3^H] thymidine incorporation assay. To analyze immune suppression specificity by ES-SCs, C3H mice were injected intraperitoneally in combination with phosphate buffered saline (PBS) or 35 Gy-irradiated ESCs-derived cells (1×10^6^). 21 days later, mice were sacrificed and spleens were harvested. CD4^+^ T cells were collected using CD4 (L3T4) MicroBeads (MiltenyiBiotec, Belgish-Bladbach). Mixed lymphocyte reaction (MLR) analysis was performed as described above. Nitric oxide (NO) concentration was estimated using Griess Reagent Kit (Promega, Fitchburg, WI).

### Measurement of NO concentration

NO in culture supernatant was detected using Griess Reagent System (Promega, WI). Analysis was performed according to the manufacturer's instructions.

### Flow cytometry and antibodies

Flow cytometry was performed using FC500 instrument (Beckman Coulter, Brea, CA) and data were analyzed by FlowJo software (Tree Star, Ashland, OR). Anti-mouse CD45 (30-F11), anti-H-2 (M1/42), anti-I-A/I-E (M5/114.15.2), anti-CD80 (16-10A1), anti-CD86 (GL1), anti-F4/80 (BM8), anti-CD11b (M1/79), anti-CD11c (HL3), anti-Gr-1 (RB6-8C5), anti-PD-L1 (MIH5), anti-PD-L2 (TY25), anti-CD115 (AFS98) and corresponding isotype controls were purchased from Biolegend (San Diego, CA) or eBioscience (San Diego, CA). For analysis, live cells were gated based on forward and side scatter as well as lack of propidium iodide uptake. All antibodies were used at 1∶100 dilutions.

### Polymerase Chain Reaction (PCR)

RNA was extracted using the RNeasy Mini Kit (Qiagen, Venlo, Netherland). 2 µg of total RNA were used for first-strand cDNA synthesis. Reverse transcription-PCR was performed using the Power SYBR Green (Life Technologies) with the following primers (all for mouse genes). *Hprt*: forward 5′-AGTCCCAGCGTCGTGATTAG-3′, reverse 5′-TCAGTCCTGTCCATAATCAGTC-3′; *Retnlα*: forward 5′-CCATAGAGAGATTATCGTGGA-3′, reverse 5′-TGGTCGAGTCAACGAGTAAG-3′; *Arginase1:* forward 5′-GTATGACGTGAGAGACCACG-3′, reverse 5′-CTCGCAAGCCAATGTACACG-3′; *Chi3l3*: forward 5′-TGGAATTGGTGCCCCTACAA-3′, reverse 5′-AACTTGCACTGTGTATATTG-3′, *Il-10:* forward 5′-GCTATGCCTGCTCTTACT-3′, reverse 5′-CCTGCTGACCTCATGATGCCA-3′, *Ho-1*: forward 5′-GATAGAGCGCAACAAGCAGAA-3′, reverse 5′-CAGTGAGGCCCATACCAGAAG-3′, *Nos2*: forward 5′- ACATCGACCCGTCCACAGTAT-3′, reverse 5′-CAGAGGGGTAGGCTTGTCTC-3′,

*Oct3/4*: forward 5′-CTCGAACCACATCCTTCTCT-3′, reverse 5′- GGCGTTCTCTTTGGAAAGGTGTTC-3′, *Tef1*: forward 5′- AAGACGTCAAGCCCTTTGTG, reverse 5′- AAAGGAGCACACTTTGGTGG, *Tbx5*: forward 5′-GGAGCCTGATTCCAAAGACA-3′, reverse 5′- TTCAGCCACAGTTCACGTTC-3′, *Mlc2v*: forward 5′- GCCAAGAAGCGGATAGAAGG-3′, reverse 5′-CTGTGGTTCAGGGCTCAGTC-3′, *αMHC*: forward 5′- GGAAGAGTGAGCGGCCATCAAGG-3′, reverse 5′- CTGCTGGAGAGGTTATTCCTCG-3′, *BNP*: forward 5′-ATGGATCTCCTGAAGGTGCT-3′, reverse 5′-TCTTGTGCCCAAAGCAGCTT-3′.

Primers for *Tgfβ1* were purchased from Qiagen.

### Transplantation

Host 129X1 or C3H mice were intraperitoneally injected with phosphate buffered saline (PBS) or 35 Gy-irradiated ESCs-derived cells (1×10^6^) 14 days before transplantation. For transplantation, embryoid bodies (EBs) of E14 ESCs or differentiated cardiomyocytes (beating EBs) were collected, re-suspended in PBS, and 3 EBs were transplanted in the host renal subcapsular region. Graft survival was monitored by direct inspection and confirmed by histological examination.

### Histological examination

Kidney samples were fixed in 4% paraformaldehyde and embedded in Tissue-Tek OCT (Sakura Finetek, Torrance, CA). 5 µm-thick sections were stained with hematoxylin and eosin. For immunohistochemical analysis, the sections were stained with anti-CD3 polyclonal antibody (N1580, Dako Japan, Tokyo), subsequently treated with a secondary antibody conjugated with biotin, and then developed using avidin-conjugated horseradish peroxidase with diaminiobenzidine. For detection of cardiac transcription factors, differentiated cardiomyocytes were plated on gelatin-coated glass coverslips at low density and fixed in acetone for 5 sec. Samples were exposed to primary antibodies using Cardiomyocyte Characterization Kit (Millipore, Darmstadt, Germany). Bound antibodies were visualized using a secondary antibody conjugated with Alexa 488. Nuclei were counterstained with 4′, 6-diamidino-2-phenylidole dihydrochloride (DAPI; Sigma Aldrich).

### Statistical Analyses

Comparisons between the groups were made with Student’s t-test, and a *P* of<0.05 was considered significant.

## Results

### Generation of immunosuppressive cells from ESCs

**Figure 1:**
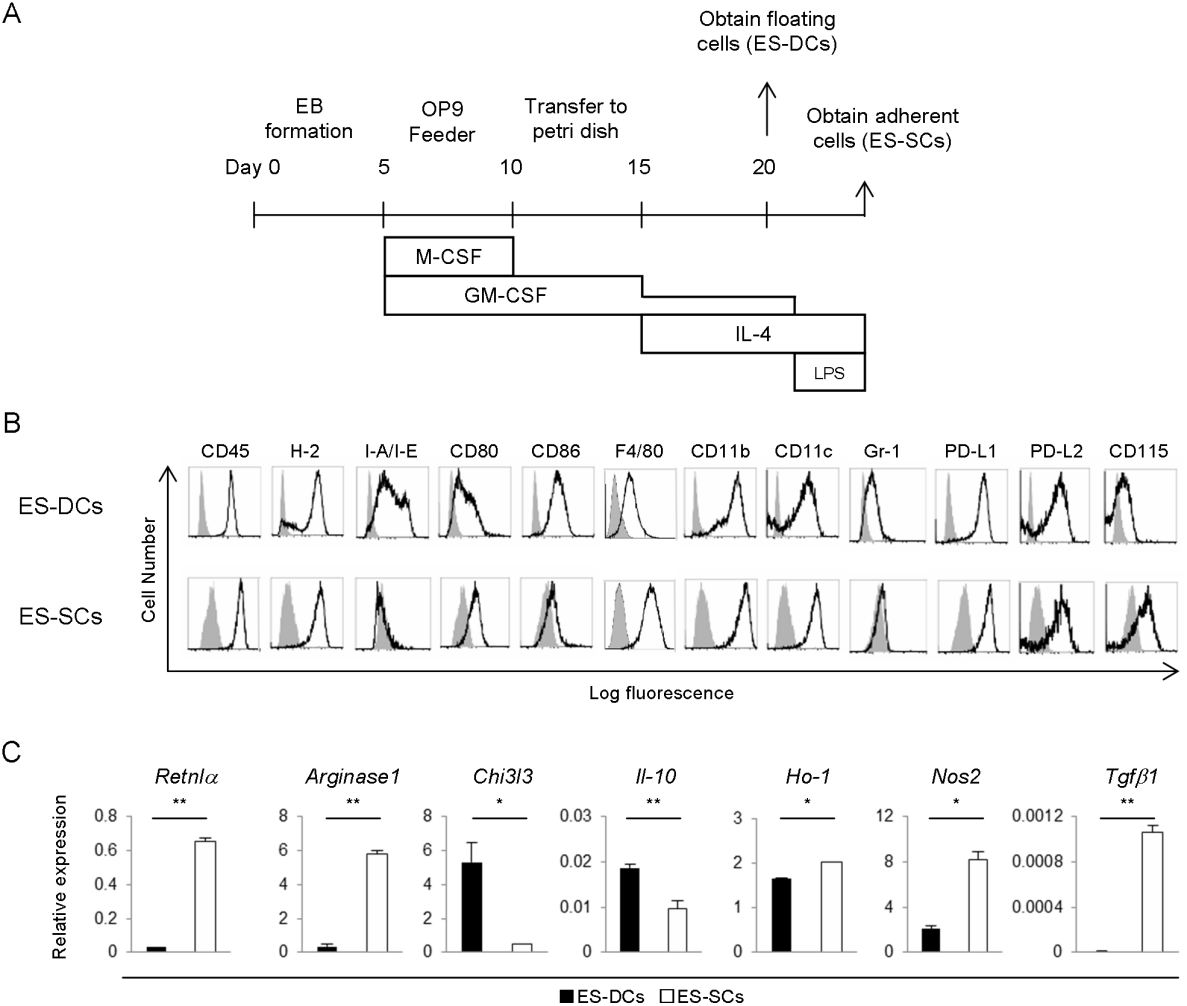
Characterization of ESCs-derived myeloid cells. (A) A scheme describes the culture protocol used to obtain floating ES-DCs and adherent ESCs-derived suppressor cells (ES-SCs). EB; embryoid body, M-CSF; macrophage colony-stimulating factor, GM-CSF; granulocyte macrophage colony-stimulating factor. (B) Flow cytometric analysis of cell surface molecular expression on ES-DCs and ES-SCs. Histogram: gray – isotype control, black line – specific antibody. Data are shown as representative of three independent experiments. (C) Quantitative RT-PCR analysis for expression of macrophage- and immunosuppression-related genes in ES-DCs and ES-SCs. Values were normalized to *Hprt* and shown as mean ± SD from three experiments were shown. *P<0.05, **P<0.01.

To generate myeloid cells from ESCs, we established a differentiation protocol based on sequential stimulation with GM-CSF, M-CSF and IL-4 (Figure 1A). Previous reports suggested that immune-stimulatory dendritic cells (DCs) could be obtained from ESCs or iPSCs with a similar protocol without using lipopolysaccharide (LPS) [14], [15]. On the other hand, other reports have shown that the addition of LPS during the process of inducing bone marrow-derived DCs resulted in the generation of immunosuppressive myeloid cells [16], [17]. However, the role of LPS stimulation in the induction of immunosuppressive cells from PSCs is unknown. Thus, we evaluated the effects of LPS stimulation on the differentiation of E14 ESCs from 129X1/SvJJmsSlc (129) mice. In the culture without LPS, we could obtain immune-stimulatory DCs (ES-DCs) in the floating cell fraction by day 20 as previously reported [14]. On the other hand, the prolonged culture with IL-4 and addition of LPS induced large, irregularly shaped cells which firmly adhered to bacteriologic petri dish surface by day 24 (Fig. 1A). To our knowledge, the adherent clusters in this culture derived from ESCs (and also from iPSCs) have not been formally investigated or reported. As shown later in this paper, these cells show substantial immunosuppressive properties, and thus we refer to them as ESCs-derived suppressor cells (ES-SCs).

### ES-SCs show immunosuppressive features comparing to ES-DCs

Next, we compared cell surface molecular expression on ES-DCs and ES-SCs by flow cytometry. As indicated in Figure 1B, major histocompatibility complex (MHC) class II (I-A/I-E), CD86 and Gr-1 expressions were lower in ES-SCs compared to ES-DCs, while F4/80 expression was higher in ES-SCs. The high expression of F4/80 suggested that ES-SCs have some characteristics of macrophages, which is consistent with their high expression of CD115 (macrophage-colony stimulating factor receptor; M-CSFR). Comparison of gene expressions between ES-DCs and ES-SCs by microarray analysis was performed and partly indicated in Figure S1A–D in File S1 (full data were deposited to Gene Expression Omnibus (http://www.ncbi.nlm.nih.gov/geo/), accession number: GSE56302). To estimate the immunosuppressive features of ES-SCs, we analyzed expression of genes which are known to correlate with T cell regulation by antigen presenting cells (Fig. S1B–D in File S1). Expression of house-keeping genes was comparable between ES-DCs and ES-SCs (Figure S1C in File S1). With few exceptions, genes which correlate with T cell inhibition were expressed higher in ES-SCs compared to ES-DCs (Fig. S1B in File S1). On the other hand, genes related to T cell stimulation were relatively expressed higher in ES-DCs than ES-SCs (Fig. S1D in File S1).

We next evaluated the expression of macrophage-related genes with real-time PCR, and found that M2 macrophage markers such as *Retnlα*, *Arginase1*, but not *Chi3l3,* were expressed higher in ES-SCs than ES-DCs (Fig. 1C). M2 macrophage subset is known to exhibit an immune-regulatory phenotype characterized by high expression of immunosuppressive molecules [18]. Thus, we next evaluated production levels of these immunosuppressive molecules in ES-DCs and ES-SCs. The expressions of *Il-10* and Heme oxygenase-1 (*Ho-1*) were not relatively different (Fig. 1C), as cell surface expressions of programmed cell death 1 ligand 1 (PD-L1) and PD-L2 were not (Fig. 1B). Interestingly, we found that ES-SCs expressed induced nitric oxide synthase (iNOS; encoded by *Nos2*) and *transforming growth factor-β1* (*Tgfβ1*) higher than ES-DCs (Fig. 1C). These results suggested that ES-SCs have immunosuppressive function rather than immunostimulatory function.

### ES-SCs Have M1/M2 macrophage hybrid phenotype

The macrophage-like features of ES-SCs was further confirmed by comparison with bone marrow-derived macrophages (BMDM) (Fig. S1E and S1F in File S1). Both ES-SCs and BMDMs showed similar morphology of spindle shaped cells which firmly adhered to plastic dishes, whereas ES-DCs could be easily detached by gentle pipetting (Fig. S1E in File S1). All three types of cells showed comparable endocytic activity (Fig. S1F in File S1). We further analyzed gene expression of ES-SCs in comparison with various subsets of bone marrow-derived macrophages described previously [19]. Gene expression of M2 markers such as *Retnlα*, *Arginase1* and *Chi3l3* were detected in ES-SCs as well as M2a cells, but not in M0 or M1 cells (Fig. S1G in File S1). *IL-10* and *Ho-1* were detected with similar levels in ES-SCs and M2a cells and relatively higher in M1 cells (Fig. S1G in File S1). *Tgfβ1*, a cytokine produced by M2 macrophages, was detected in comparable levels among ES-SCs, M1 and M2 cell. As mentioned above, ES-SCs also showed expression of *Nos2*, one of the M1 markers (Fig. S1G in File S1).

Microarray data were further analyzed focusing on macrophage markers in ES-SCs compared to bone marrow derived-M0, M1, M2a, M2b, M2c and Mreg (Fig. S1H–N in File S1). Gene expression of ES-SCs was relatively correlated with M1 cells (r^2^ = 0.2981) than other types of macrophages in this comparison. On the other hand, M2 markers such as *Chi3l3* and *Retnlα* were highly expressed in ES-SCs as well as M2a cells. Together, these results suggest that ES-SCs resemble macrophages in morphology and phagocytic capacity, having M1/M2 macrophage hybrid phenotype, and express some immunosuppressive genes such as *Nos2* and *Tgfβ1* [20], [21].

### ES-SCs display immune-regulatory functions

**Figure 2:**
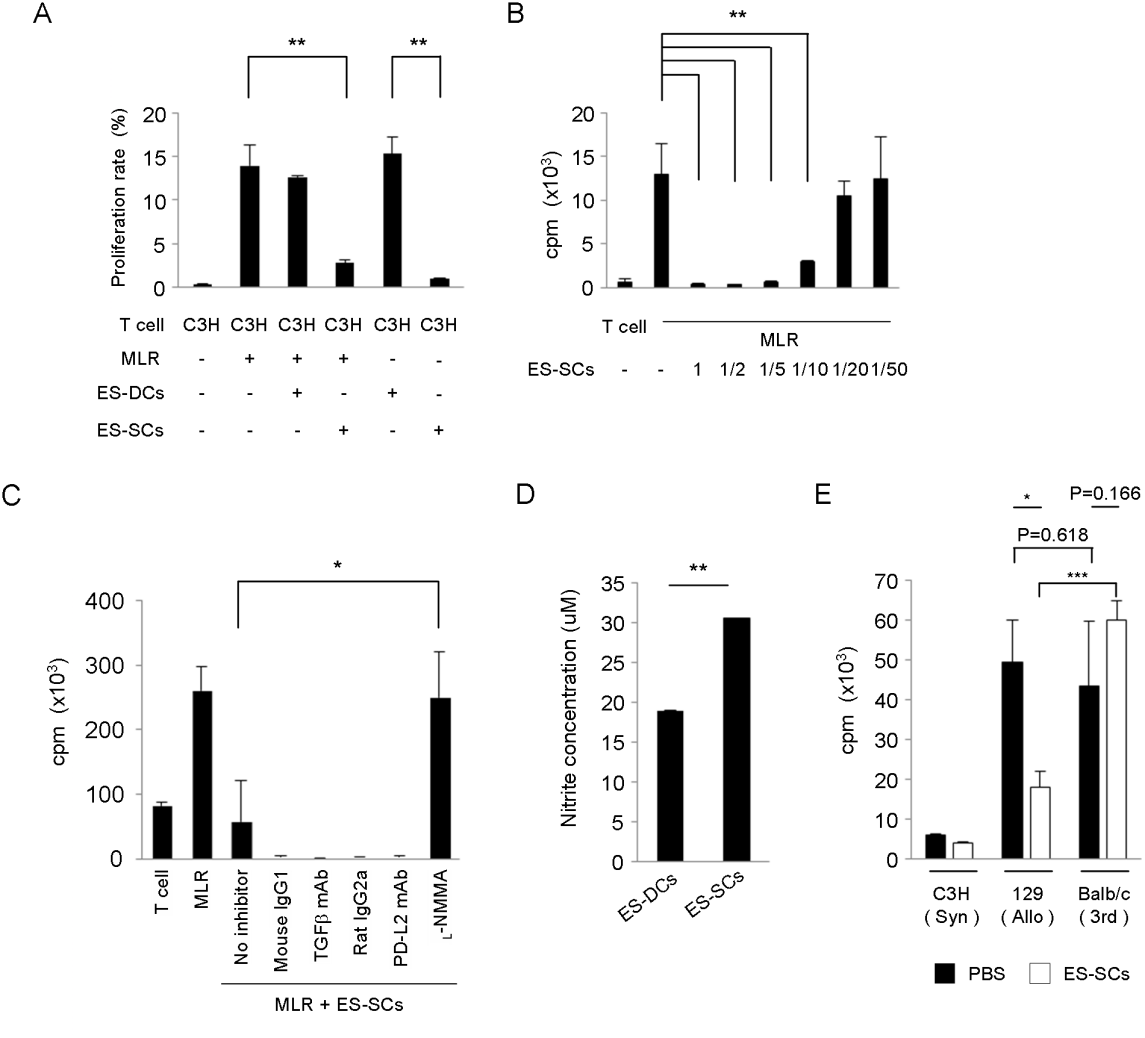
ES-SCs suppress T cell proliferation. (A) MLR assay: C3H T cells vs. bone marrow-derived DCs. Where indicated, 129 ES-DCs or ES-SCs were added to MLR culture and inhibitory effects were measured by proliferation rate (CFSE cell division). (B) ES-SCs and MLR assay: C3H T cells were stimulated with irradiated bone marrow-derived DCs, and then graded numbers of ES-SCs were added to MLR culture. T cell proliferation was estimated by [^3^H] thymidine uptake. Results are expressed as mean cpm ± SD. (C) ES-SCs and MLR assay. Several immunosuppressive molecules were blocked by specific inhibitors: Anti-TGFβ mAb (10 µg/mL), anti-PD-L2 mAb (10 µg/mL), _L_-NMMA (50 µM), or corresponding isotype-matched controls were added. T cell proliferation was estimated by [^3^H] thymidine uptake. Results are expressed as mean cpm ± SD. (D) Measurement of NO concentration in the supernatants of ES-DCs or ES-SCs. (E) Specificity of immune suppression by ES-SCs. Splenic T cells from ES-SCs- or PBS-administered C3H mice were stimulated with bone marrow-derived DCs from the indicated mice including C3H (Syn; Syngeneic), 129 (Allo; allogeneic but same background as ES-SCs), and BALB/c (3rd; allogeneic, third party). T cell proliferation was estimated by [^3^H] thymidine uptake. Data represent mean ± SD of three wells. Similar results were obtained from three independent experiments. *P<0.05, **P<0.01, ***P<0.001.

As we anticipated obtaining immune-regulatory cells from PSCs, we next examined whether either ES-DCs or ES-SCs could interfere with allogeneic immune response. To do so, we stimulated T cells isolated from C3H/HeSlc (C3H; H-2^k^) mice with allogeneic bone marrow-derived DCs of 129 mice (H-2^b^) (mixed lymphocyte reaction; MLR) in the presence or absence of either ES-DCs or ES-SCs. We found that the addition of ES-SCs significantly inhibited T cell response, whereas ES-DCs were almost ineffective (Fig. 2A). To examine antigen-presenting capacity of the cells, ES-SCs or ES-DCs were used as stimulators of MLR instead of bone marrow-derived DCs. Interestingly; a significant proliferation of C3H T cells was observed when co-cultured with ES-DCs but not ES-SCs (Fig. 2A). Importantly, ES-SCs could remarkably inhibit T cell proliferation at a low ratio of 1/10: ES-SCs/T cell (Fig. 2B). These results indicate that ES-SCs, but not ES-DCs, possess a capacity to inhibit allogeneic T cell responses.

To explore possible mechanisms of ES-SCs-mediated immune suppression, we then blocked several immunosuppressive candidate molecules and evaluated the consequences on T cell proliferation. We set MLR in which ES-SCs inhibited the proliferation of allogeneic T cell (no inhibitor, Fig. 2C). We found that the blockade of TGFβ or PD-L2 with corresponding monoclonal antibody could not restore allogeneic T cell proliferation (Fig. 2C). On the other hand, inhibition of iNOS function with _L_-NG-monomethyl Arginine citrate (_L_-NMMA), an inhibitor that reduces nitric oxide (NO) production, significantly restored T cell proliferation (Fig. 2C). According with this, concentration of NO in the supernatant of ES-SCs was higher than that of ES-DCs (Fig. 2D). Therefore, we concluded that, at least partially, iNOS-mediated NO production from ES-SCs could be responsible for the suppression of allogeneic T cell proliferation.

### Regulatory T cells are not involved in immunosuppressive function of ES-SCs

In addition to ES-SCs, we have previously reported the ability to generate regulatory T cells (Tregs) from ESCs or iPSCs, which have the potential to suppress T cell responses [22]. Thus, we next confirmed if Tregs were generated in the differentiation protocol used in this study. As shown in Figure S2A in File S1, ES-SCs showed no expression of T cell markers such as CD3, CD4, CD8 or *α*β-T cell receptor. Furthermore, we analyzed the capacity of ES-SCs to induce Tregs. To do so, CD4^+^ T cells were isolated from splenocytes and cultured in anti-CD3 antibody-coated plate in the presence of TGFβ1 and IL-2 to induce Tregs differentiation, or co-cultured with ES-SCs. The fraction of CD4^+^CD25^+^FoxP3^+^ T cells efficiently increased in the standard induction protocol but not in ES-SCs co-culture or untreated cells (Fig. S2B in File S1). These results suggested that Tregs were not generated or induced in ES-SCs culture, and thus were not involved in immunosuppressive phenotype of ES-SCs.

### ES-SCs induce immune suppression in alloantigen-specific manner

We then examined whether ES-SCs induce immune suppression in the experiment described above in an alloantigen-specific manner. C3H mice were injected with 35 Gy-irradiated ES-SCs (1×10^6^ cells) or PBS. 21 days later, splenic T cells were purified and their reactivity against bone marrow-derived DCs of C3H mice (syngeneic), 129 mice (allogeneic, same background as ES-SCs, H-2^b^), or BALB/c mice (allogeneic, third party, H-2^d^) was evaluated by T cell proliferation assay (Fig. 2E). T cells derived from PBS-treated mice proliferated well by stimulation with DCs of 129 and BALB/c mice. Consistent with the results shown in Table 1 and 2, T cells from ES-SCs-treated mice proliferated minimally by stimulation with DCs of 129 as well as C3H mice. On the other hand, T cells from ES-SCs-treated mice significantly proliferated when stimulated with DCs of BALB/c mice. These results suggest that ES-SCs induce immune suppression in a donor antigen specific manner (in this case, H-2^b^).

**Table 1:** Graft (ESCs) survival.

Recipients (Treatment)	Transplanted grafts	Day14 (%Survival)	Day21 (%Survival)	Day28 (%Survival)
129 (PBS)	E14 ESCs	12/12 (100)	n.d.	10/10^a^ (100)
C3H (PBS)	E14 ESCs	0/7 (0)	-	-
C3H (ES-SCs)	E14 ESCs	7/7 (100)	n.d.	0/5^a^ (0)
C3H (ES-DCs)	E14 ESCs	0/7 (0)	-	-

Administration of ES-SCs prolonged allogeneic ESCs-derived embryoid body-like sphere survival *in vivo*. Recipient mice were pretreated with PBS, ES-SCs or ES-DCs as described in Materials and Methods. Thereafter, mice received transplantation of embryo body-like sphere generated from 129-derived ESCs, and graft survival was monitored by direct inspection. C3H (PBS-injected) vs. C3H (ES-SCs-treated), p<0.001; Generalized Wilcoxon’s test.

a Several recipient mice were sacrificed for histological examination.

n.d., not determined.

**Table 2:** Graft (ESCs-derived cardiomyocytes) survival.

Recipients (Treatment)	Transplanted grafts	Day14 (%Survival)	Day21 (%Survival)	Day28 (%Survival)
129 (PBS)	ESCs-derived cardiomyocytes	9/9 (100)	7/7^a^ (100)	7/7 (100)
C3H (PBS)	ESCs-derived cardiomyocytes	3/10 (30)	2/10 (20)	1/10 (10)
C3H (ES-SCs)	ESCs-derived cardiomyocytes	9/10 (90)	6/8^a^ (75)	5/8 (63)
C3H (ES-DCs)	ESCs-derived cardiomyocytes	0/10 (0)	-	-

Administration of ES-SCs prolonged allogeneic ESCs-derived cardiomyocytes survival *in vivo* ***.*** Recipient mice were pretreated by PBS, ES-SCs or ES-DCs as described in Materials and Methods. Thereafter, mice received transplantation of cardiomyocytes generated from 129-derived ESCs, and graft survival was monitored by direct inspection. C3H (PBS-injected) vs. C3H (ES-SCs-treated), p<0.01; Generalized Wilcoxon’s test.

a Several recipient mice were sacrificed for histological examination.

### ES-SCs prolonged allogeneic graft survival

**Figure 3:**
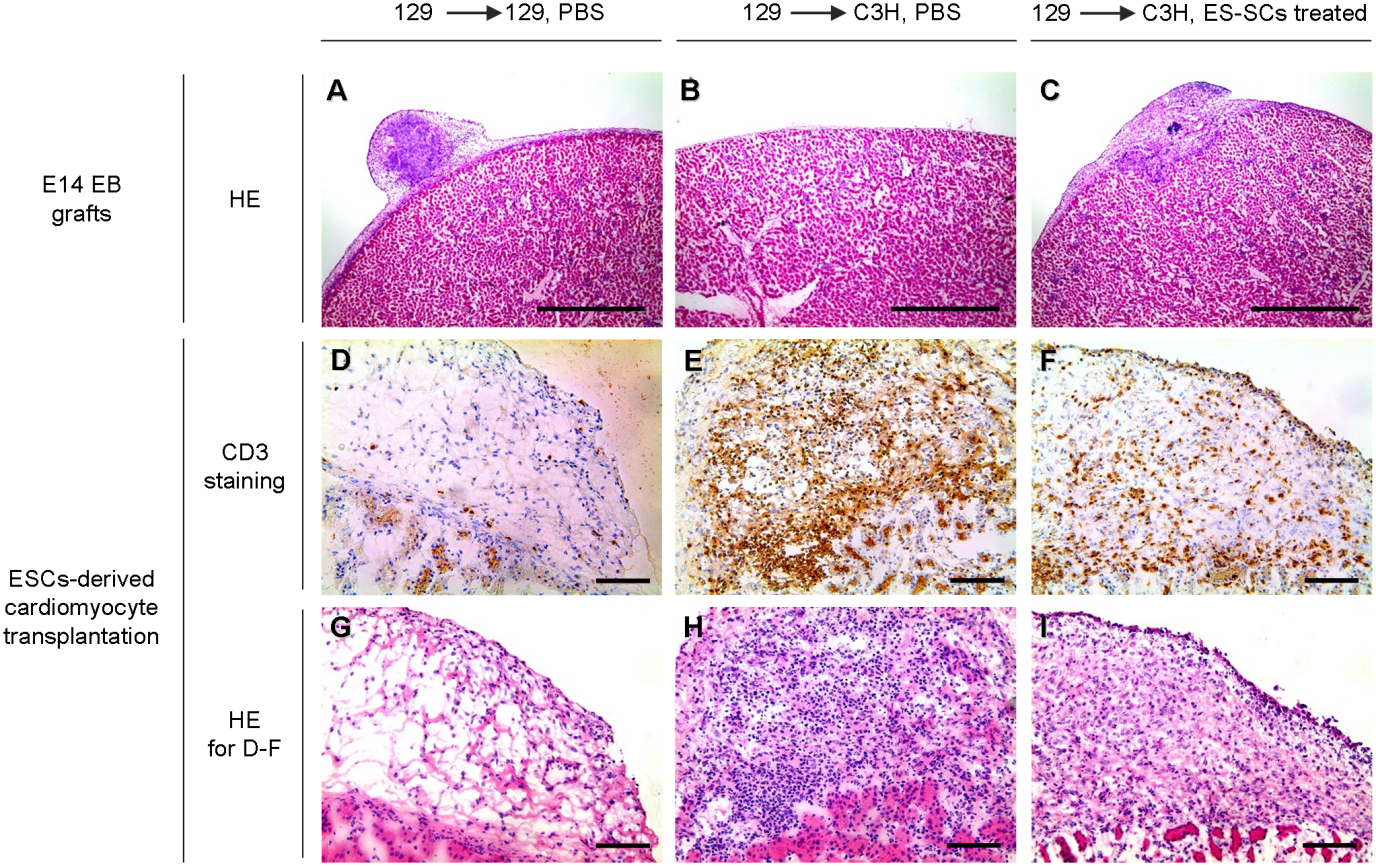
ES-SCs prolonged the survival of allogeneic graft. Host 129X1 or C3H mice were intraperitoneally injected with phosphate buffered saline (PBS, Figure 3A, B, D, E, G and H) or 35 Gy-irradiated ES-SCs (Figure 3C, F and I) 14 days before transplantation. (A–C) Hematoxylin and eosin staining of E14 EB grafts transplanted into renal subcapsule at day 14 after graft transplantation. Data are shown as representative of two grafts. Scale bars, 1.0 mm. (D–F) Immunohistological staining for CD3 antigen (indicating T cell infiltration) of ESCs-derived cardiomyocytes transplanted into renal subcapsule at day 7 after graft transplantation. (G–I) Hematoxylin and eosin staining of ESCs-derived cardiomyocytes transplanted into renal subcapsule at day 7 after graft transplantation. Data are shown as representative of two grafts. Scale bars, 100 µm.

Next, we evaluated the effects of ES-SCs on the suppression of allogeneic T cell response in *in vivo* allogeneic transplantation model. 129 or C3H mice were injected with phosphate buffered saline (PBS) or 35 Gy-irradiated ES-SCs or ES-DCs (1×10^6^ cells). 14 days later, embryoid bodies (EB) derived from E14 ESCs were transplanted into renal subcapsular region of the mice. Graft survival was determined by direct macroscopic inspection of teratoma formation at day 14 and 28 after the transplantation (Fig. S3 in File S1). As indicated in Table 1, syngeneic grafts grew normally at day 28, whereas no allogeneic untreated grafts were detectable even at day 14. On the other hand, allogeneic grafts in recipients that were pretreated with ES-SCs, remarkably survived at least until day 14, but were not detectable at day 28. Pretreatment with ES-DCs did not prolong the graft survival. Microscopic analysis at day 14 confirmed the graft acceptance in syngeneic, allogeneic and ES-SCs-pretreated recipients (Figs. 3A–C). These results suggested that the treatment with ES-SCs has a potential to improve the outcomes of allogeneic transplantation.

Finally, we examined whether ES-SCs could prolong the survival of normally differentiated grafts derived from ESCs. For this purpose, we induced cardiomyocytes from E14 ESCs by utilizing Noggin according to a previous report [12]. This protocol efficiently induced mRNA expression of cardiac transcription factors, including *Tef1* and *Tbx5,* whereas expression of a stem cell marker *Oct3/4* rapidly decreased by day 7 (Fig. S4A in File S1). On day 14, expression of cardiac-specific molecules such as Troponin I, Actinin, Tropomyosin, or arterial natriuretic peptide (ANP) were detected by immunostaining (Fig. S4B in File S1). The differentiated cells formed beating spheres (thus we called them beating EBs), and were grafted under the kidney capsule of syngeneic 129 or allogeneic C3H mice which had been pretreated with PBS or ES-SCs. Again in this experiment, we observed that pretreatment with ES-SCs in allogeneic recipients decreased CD3^+^ cellular infiltration (Figs. 3D–I) and substantially prolonged the graft survival (Table 2). On the other hand, pretreatment with ES-DCs was ineffective to prolong the graft survival (Table 2).

To evaluate the contribution of ES-SCs at the site of beating EBs transplantation, transplanted grafts were examined by immuno-fluorescence staining for T cells and macrophage markers (Figure S5A and B in File S1). Consistent with Figure 3D–F, allogeneic grafts were highly infiltrated with CD3^+^ T cells compared to syngeneic grafts that were negative for CD3 staining. However, pretreatment with ES-SCs was effective to reduce CD3^+^ T cells infiltration into transplanted allogeneic grafts (Figure S5A in File S1). F4/80 staining revealed the existence of F4/80^+^ cells in all cases (Figure S5A in File S1). To identify the origin of these F4/80^+^ cells, double staining by H-2D^b^ and F4/80 was performed (Figure S5B in File S1). H-2D^b^ is one of MHC class 1 molecule which is expressed in some strains of mice including 129 mice but not C3H mice. Thus, H-2D^b^ serves as an indicator for 129X1-derived ES-SCs (Figure 1B). As expected, in syngeneic transplantation (without ES-SCs pretreatment), F4/80 positive cells were also H-2D^b^ positive (Figure S5B in File S1). On the other hand, H-2D^b^ expression in F4/80^+^ cells was not detected in cases of allogeneic transplantation and allogeneic transplantation with ES-SCs pretreatment. (Figure S5B in File S1). Thus, these results suggest that ES-SCs might not act directly at local sites of transplantation, but have the potential to improve the outcomes of allogeneic transplantation of ESCs-derived grafts.

## Discussion

In the last century, organ transplantation has been progressed with the development of several excellent immunosuppressants including calcineurin inhibitors [4]. Furthermore, a lot of regimens based on the manipulation of molecules expressed in immune cells have been reported to suppress allogeneic immune responses. For example, blockade of costimulatory molecules such as CD28 or CD154 with monoclonal antibodies has been shown to prolong allogeneic graft survival [23], [24], and combinations of hematopoietic stem cell transplantation with T cell inhibition were effective to induce indefinite graft survival [10], [25]. When PSCs such as ESCs or others derived iPSCs are used as a source of cells or tissues for transplantation, these kinds of immune manipulation should be considered. In this study, we have examined whether immune regulatory cells could be induced from ESCs. In the present study, we have shown that macrophage-like immunosuppressive cells could be differentiated from mouse ESCs (i.e., ES-SCs) that contribute to prolong allogeneic graft survival.

Besides ES-SCs, we have previously shown that Foxp3-positive regulatory T cells (Tregs) can be induced from ESCs or iPSCs [22]. Tregs derived from PSCs have a potential to suppress activation of other T cells. Therefore, ESCs- or iPSCs-derived Tregs can also be one of the candidates for cell-based immune regulation in PSCs-using cell replacement therapy. However, the efficacy of Tregs generation from PSCs appeared to be lower than that of ES-SCs (1 in 100^th^∼1000^th^, data not shown). Furthermore, it was difficult to obtain pure Tregs from differentiation culture of ESCs or iPSCs [22]. Compared to PSCs-derived Tregs, it was relatively easy to obtain ES-SCs as adherent fraction from the myeloid cell differentiation. The obtained ES-SCs seemed mostly homogeneous, as judged by the cell surface molecule expression shown in this study. However, attempts to further purify the immunosuppressive fraction may improve the transplant results. In this context, we have examined detailed gene expression in ES-SCs with microarray analysis. This analysis suggested that several molecules such as Cx3cr1 (Figure S1A in File S1) might be specifically expressed in the adherent fraction and thus useful to divide it into subpopulations. Our current works focus on further investigations with such information that may enable us to identify a sorting marker(s) for obtaining pure ES-SCs from the adherent fraction.

Tissue macrophages are known to have the ability to differentiate into classically (M1) or alternatively (M2) activated macrophages [21]. In ES-SCs, typical signature genes for M2 macrophages like *Retnl*α (encoding Relm-α/Fizz1) or *Arginase1* (encoding Arginase1) were remarkably up-regulated. M2-biased macrophages exposed to IL-4 have been shown to acquire an inhibitory activity against T cells [26]. On the other hand, iNOS expression is considered as one of molecular markers for M1 macrophages, by which L-arginine is metabolized into L-citrulline and NO. It is also known that LPS induces up-regulation of iNOS in macrophages [26]. Therefore, we considered that ES-SCs had M1/M2 hybrid phenotype and exerted immune-regulatory capacity via iNOS expression.

A previous study has reported the ability to induce M2-like macrophages from ESCs [27]. ES-SCs and ESCs-derived M2-like macrophages seem to be different, as they were induced in the absence of IL-4, showing reduced responsiveness to LPS and unexpected pro-inflammatory features [27]. In contrast, ES-SCs seem to rather resemble another population of macrophages which were induced from bone marrow with IFN-γ treatment [28]. The IFN-γ treatment-induced macrophages expressed iNOS and significantly inhibited allogeneic transplant rejection via iNOS-mediated mechanisms [28], [19]. Stimulation of ES-SCs with LPS in the presence of IL-4 in our protocol might work as IFN-γ treatment reported by Brem-Exner, et al.

Regarding the generality of culture protocol shown in this study, we could induce similar cells to ES-SCs from both mouse and human iPSCs (manuscript in preparation). Thus, this protocol is capable to obtain immune-regulatory cells from PSCs of mice and humans. Furthermore, the ES-SCs-like immunosuppressive cells derived from mouse iPSCs inhibited allogeneic transplant rejection (manuscript in preparation). These results are clinically relevant; because not only ESCs but also iPSCs would be subjects of allogeneic immune rejection when used for donor source of cell replacement therapy. As to the suppression mechanism of the induced human macrophage-like cells, TGFβ but not iNOS would be more important, because there has been a theory that the latter is barely produced from human macrophages [29]. Whatever type of immunosuppressive cells are induced from PSCs, similar strategies would be useful for the development of safe and effective immunotherapy for cell replacement therapy based on PSCs.

## Supporting Information

File S1 **Combined file of supporting figures. Figure S1, related to** **Figure 1** **. Additional data for characterization of ES-SCs.** (A) Heat maps from microarray analysis between ES-DCs and ES-SCs. (B–D) Gene expression analysis by microarray data between ES-SCs and ES-DCs were shown in scatter plots and heat maps. The expression of genes related to immunosuppressive function (B), house keeping (C) and T cell activation (D) was plotted according to its signal intensity. Dotted line is an auxiliary, indicates same expression level between ES-SCs and ES-DCs. (E) Morphology of ES-DCs, ES-SCs and bone marrow-derived macrophages (BMM). Scale bars, 100µm. (F) Endocytosis assay. Fluorescence-labeled *Staphylococcus aureus* (*S. aureus*) or *Escherichia coli* (*E. coli*) particles were incubated with the indicated cells. After incubation, cells were analyzed for the increase of cell-associated fluorescence (filled histograms). Open histograms represent untreated cells. (G) Gene expression analysis of bone marrow-derived macrophages (M0, M1 and M2a) and ES-SC. M0, M1 and M2a cells were generated as previously described (Riquelme P. *et al*., *Mol. Ther*. 21(2), 409–422.2013). Values are normalized to *Hprt* and shown as mean ± SE (*p<0.05, **p<0.01, unpaired Student’s t test). The expression of genes related to M1, M2 and macrophage markers were analyzed based on microarray data between ES-SCs and bone marrow macrophages (data from Riquelme et al., GEO Series accession number: GSE32690). To normalize the data between Riquelme et al.’s data and our data, signal intensity of ES-SCs were normalized by following formula: Adjusted value = Log_2_{(Original signal intensity value)^0.5^}. The results were shown in heat maps (H) and scatter plots (I–N). Linear regression was performed to evaluate the gene expression similarity between ES-SCs and macrophages (M0, M1, M2a, M2b, M2c and Mreg). R^2^ values shown on the top of each figures (I–N). **Figure S2. Unlike contribution of regulatory T cells.** (A) ES-SCs were stained by mAbs for T cells markers, and analyzed using flow cytometer (Upper panel). Splenocytes were used as positive control (Lower Panel). (B) Capacity of regulatory T cell induction. CD4^+^ T cells were isolated from splenocytes and cultured for 72 hours in anti-CD3 mAb-coated plate with TGFβ1 and IL-2 (Top column, positive control), medium alone (middle column, negative control) or co-cultured with ES-SCs (bottom column). Generated cells were analyzed for the expression of FoxP3 and CD4 or CD25 using flow cytometry. **Figure S3, related to** **Table 1** **and** **Table 2** **. Photography of the grafts transplanted under kidney capsules.** The arrows indicate the transplanted sites. After taking these photos, the incisions were closed, and the recipient mice were kept alive until at least day 28. **Figure S4, related to** **Fig. 3** **and** **Table 1** **and** **2** **.** (A) Expression of stem cell marker and cardiac transcription factors in ESCs and ES-SCs detected by RT-PCR. Representative data from two experiments. (B) Immunostaining for Troponin I, Actinin, Tropomyosin and ANP. ESCs-derived cardiomyocyes were attached to gelatin-coated tissue culture plate and stained with the cardiomyocyte-specific antibodies. All the samples were counter-stained with DAPI. Representative data from three experiments. Scale bars, 100 µm. **Figure S5, ES-SCs act systemically rather than locally at transplantation sites.** Immunohistological staining for CD3 antigen (indicating T cell infiltration) of ESCs-derived cardiomyocytes transplanted into renal subcapsule at day 6 after transplantation. Recipient mouse was pre-treated with PBS (129→129, PBS and 129→C3H, PBS) or ES-SCs (129→C3H, ES-SCs treated). Dotted line indicated the border of kidney and graft. g; graft, k; kidney. Scale bars, 100 µm. *non-specific staining of kidney cells. (A) Immunohistological staining for CD3 antigen (indicating T cell infiltration) or F4/80 antigen (indicating macrophage infiltration). (B) Immunohistological staining for F4/80 antigen and H-2D^b^ antigen (indicating recipient cell infiltration in recipient 129 mouse or ES-SCs in recipient C3H mouse).
(PPT)

Materials and Methods S1 (PPT)
