# Supplementary material for: Induction of Macrophage-Like Immunosuppressive Cells from Mouse ES Cells That Contribute to Prolong Allogeneic Graft Survival
Source: PLoS One. 2014 Oct 30;9(10):e111826. doi: 10.1371/journal.pone.0111826 (PMC4214817; doi:10.1371/journal.pone.0111826)
Supplement: File S1 — Combined file of supporting figures. Figure S1, related to Figure 1 . Additional data for characterization of ES-SCs. (A) Heat maps from microarray analysis between ES-DCs and ES-SCs. (B–D) Gene expression analysis by microarray data between ES-SCs and ES-DCs were shown in scatter plots and heat maps. The expression of genes related to immunosuppressive function (B), house keeping (C) and T cell activation (D) was plotted according to its signal intensity. Dotted line is an auxiliary, indicates same expression level between ES-SCs and ES-DCs. (E) Morphology of ES-DCs, ES-SCs and bone marrow-derived macrophages (BMM). Scale bars, 100µm. (F) Endocytosis assay. Fluorescence-labeled Staphylococcus aureus (S. aureus) or Escherichia coli (E. coli) particles were incubated with the indicated cells. After incubation, cells were analyzed for the increase of cell-associated fluorescence (filled histograms). Open histograms represent untreated cells. (G) Gene expression analysis of bone marrow-derived macrophages (M0, M1 and M2a) and ES-SC. M0, M1 and M2a cells were generated as previously described (Riquelme P. et al., Mol. Ther. 21(2), 409–422.2013). Values are normalized to Hprt and shown as mean ± SE (*p<0.05, **p<0.01, unpaired Student’s t test). The expression of genes related to M1, M2 and macrophage markers were analyzed based on microarray data between ES-SCs and bone marrow macrophages (data from Riquelme et al., GEO Series accession number: GSE32690). To normalize the data between Riquelme et al.’s data and our data, signal intensity of ES-SCs were normalized by following formula: Adjusted value = Log2{(Original signal intensity value)0.5}. The results were shown in heat maps (H) and scatter plots (I–N). Linear regression was performed to evaluate the gene expression similarity between ES-SCs and macrophages (M0, M1, M2a, M2b, M2c and Mreg). R2 values shown on the top of each figures (I–N). Figure S2. Unlike contribution of regulatory T cells. (A) ES-SCs were staine [file pone.0111826.s001.ppt]

## Slide 1
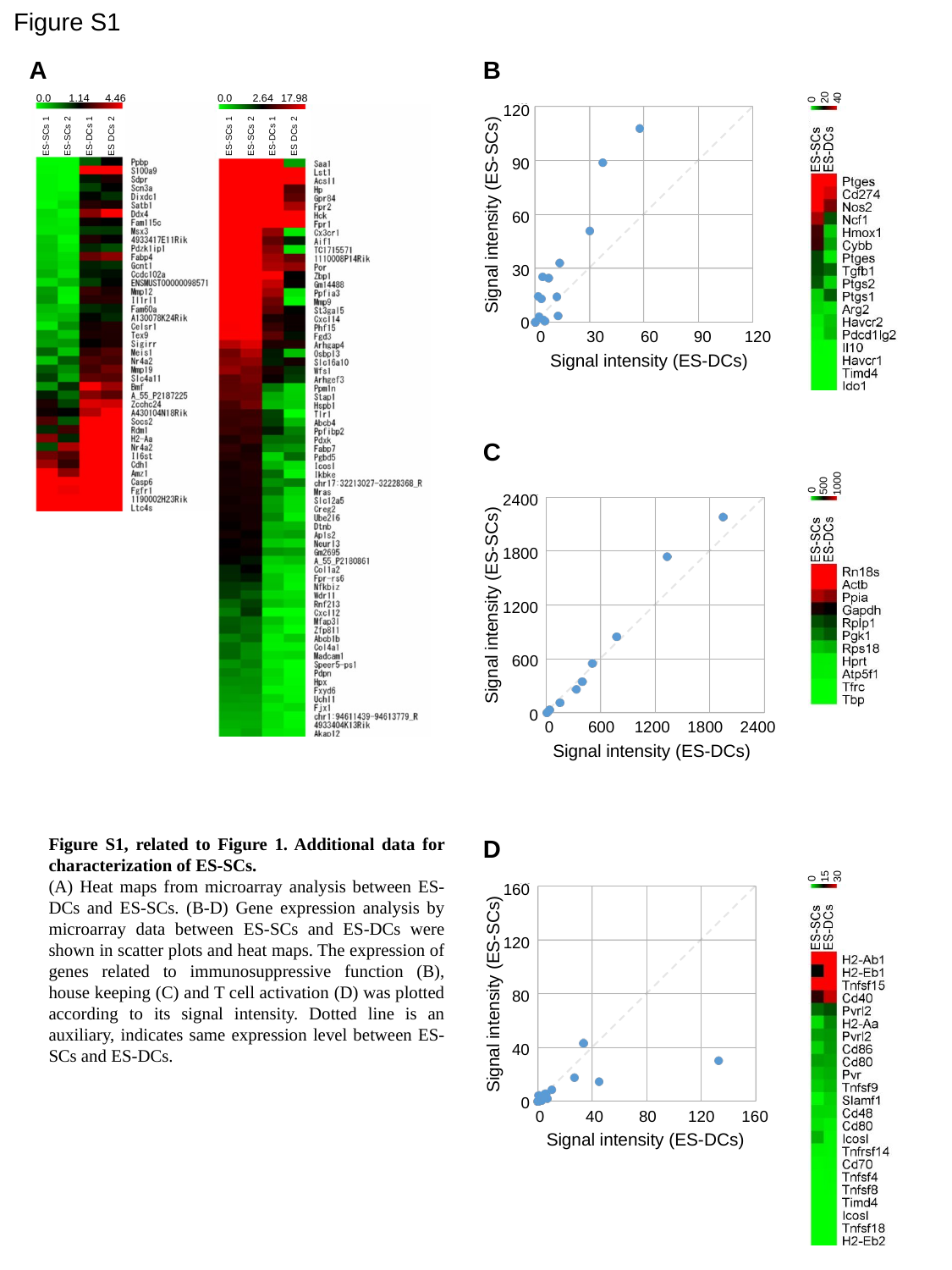

Figure S1
A
B
0.0
1.14
4.46
0.0
2.64
17.98
20
40
0
120
90
60
30
0
Signal intensity (ES-SCs)
0
30
60
90
120
Signal intensity (ES-DCs)
ES-DCs 1
ES-DCs 1
ES-SCs 1
ES-SCs 2
ES DCs 2
ES-SCs 1
ES-SCs 2
ES DCs 2
C
1000
500
0
2400
1800
Signal intensity (ES-SCs)
1200
600
0
0
600
1200
1800
2400
Signal intensity (ES-DCs)
Figure S1, related to Figure 1. Additional data for characterization of ES-SCs.
(A) Heat maps from microarray analysis between ES-DCs and ES-SCs. (B-D) Gene expression analysis by microarray data between ES-SCs and ES-DCs were shown in scatter plots and heat maps. The expression of genes related to immunosuppressive function (B), house keeping (C) and T cell activation (D) was plotted according to its signal intensity. Dotted line is an auxiliary, indicates same expression level between ES-SCs and ES-DCs.
D
15
30
0
160
120
80
40
0
Signal intensity (ES-SCs)
0
40
80
120
160
Signal intensity (ES-DCs)

## Slide 2
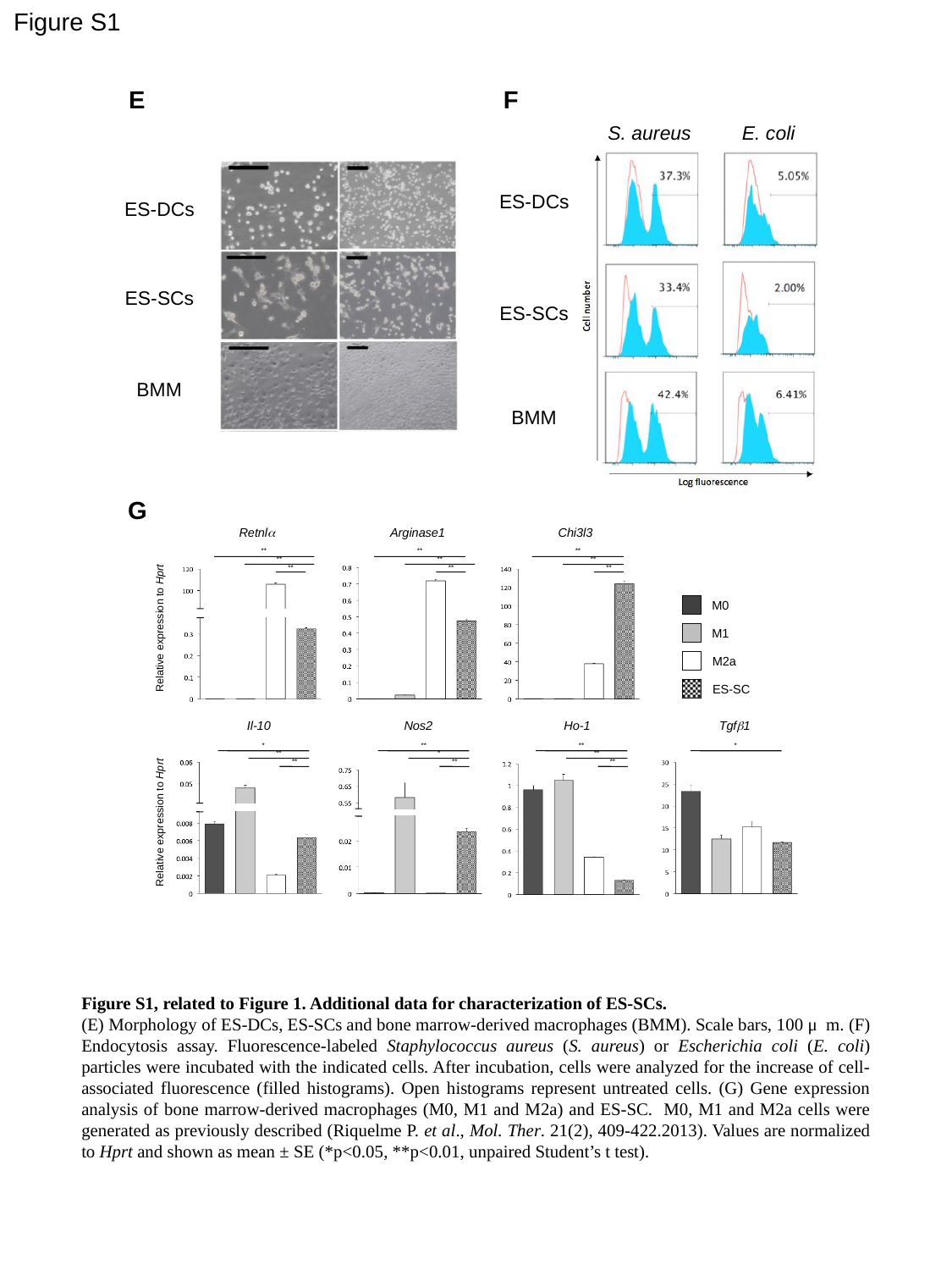

Figure S1
E
F
S. aureus
E. coli
ES-DCs
ES-DCs
ES-SCs
ES-SCs
BMM
BMM
G
Retnlα
Arginase1
Chi3l3
**
**
**
**
**
**
**
**
**
M0
Relative expression to Hprt
M1
M2a
ES-SC
Il-10
Nos2
Ho-1
Tgfβ1
*
**
**
**
*
**
**
**
**
*
Relative expression to Hprt
Figure S1, related to Figure 1. Additional data for characterization of ES-SCs.
(E) Morphology of ES-DCs, ES-SCs and bone marrow-derived macrophages (BMM). Scale bars, 100 μ m. (F) Endocytosis assay. Fluorescence-labeled Staphylococcus aureus (S. aureus) or Escherichia coli (E. coli) particles were incubated with the indicated cells. After incubation, cells were analyzed for the increase of cell-associated fluorescence (filled histograms). Open histograms represent untreated cells. (G) Gene expression analysis of bone marrow-derived macrophages (M0, M1 and M2a) and ES-SC. M0, M1 and M2a cells were generated as previously described (Riquelme P. et al., Mol. Ther. 21(2), 409-422.2013). Values are normalized to Hprt and shown as mean ± SE (*p<0.05, **p<0.01, unpaired Student’s t test).

## Slide 3
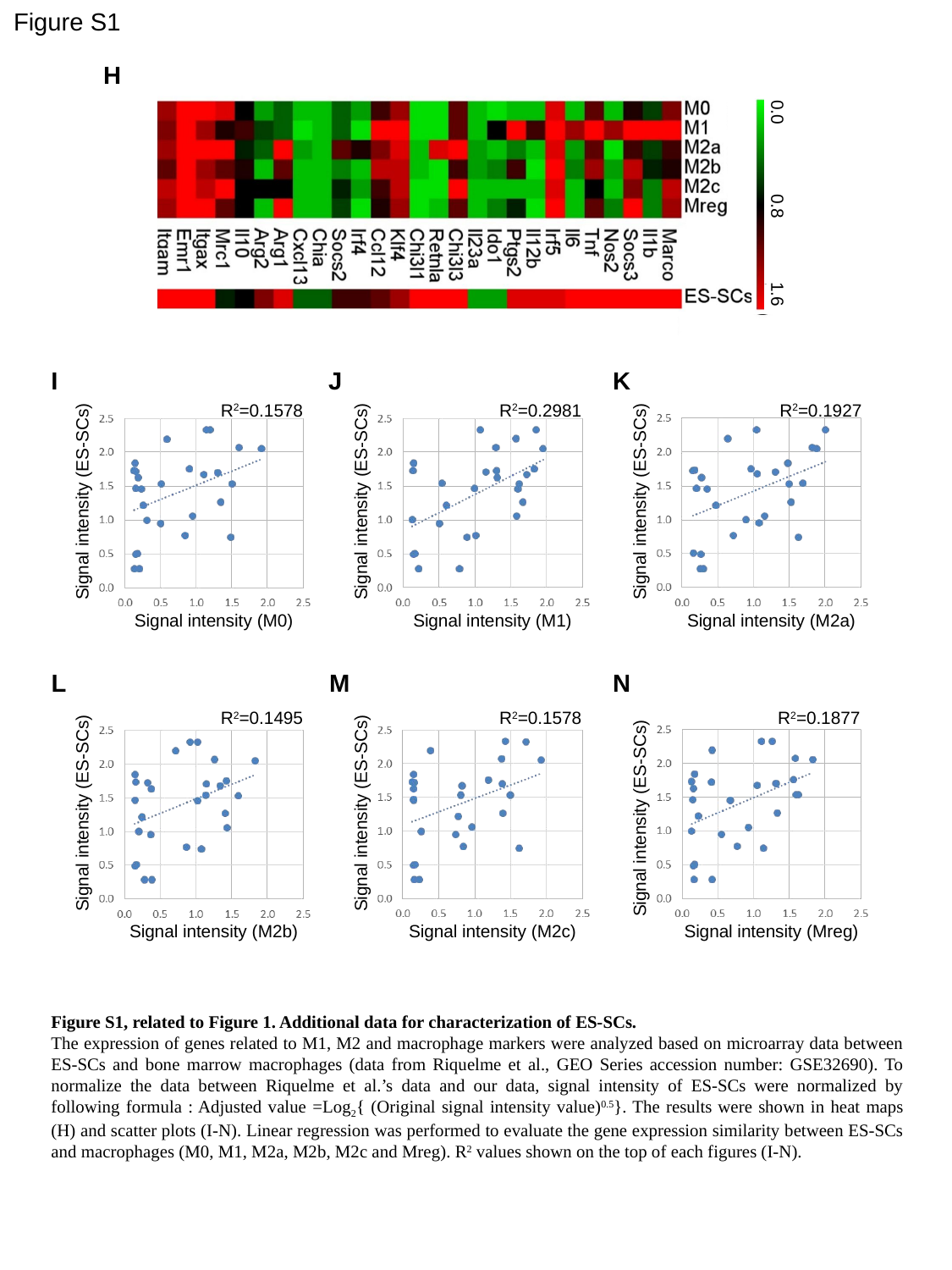

Figure S1
H
0.0
0.8
1.6
I
J
K
Signal intensity (ES-SCs)
Signal intensity (M0)
Signal intensity (ES-SCs)
Signal intensity (M1)
Signal intensity (ES-SCs)
Signal intensity (M2a)
R2=0.1578
R2=0.2981
R2=0.1927
L
M
N
R2=0.1495
R2=0.1578
R2=0.1877
Signal intensity (ES-SCs)
Signal intensity (ES-SCs)
Signal intensity (ES-SCs)
Signal intensity (M2b)
Signal intensity (M2c)
Signal intensity (Mreg)
Figure S1, related to Figure 1. Additional data for characterization of ES-SCs.
The expression of genes related to M1, M2 and macrophage markers were analyzed based on microarray data between ES-SCs and bone marrow macrophages (data from Riquelme et al., GEO Series accession number: GSE32690). To normalize the data between Riquelme et al.’s data and our data, signal intensity of ES-SCs were normalized by following formula : Adjusted value =Log2{ (Original signal intensity value)0.5}. The results were shown in heat maps (H) and scatter plots (I-N). Linear regression was performed to evaluate the gene expression similarity between ES-SCs and macrophages (M0, M1, M2a, M2b, M2c and Mreg). R2 values shown on the top of each figures (I-N).

## Slide 4
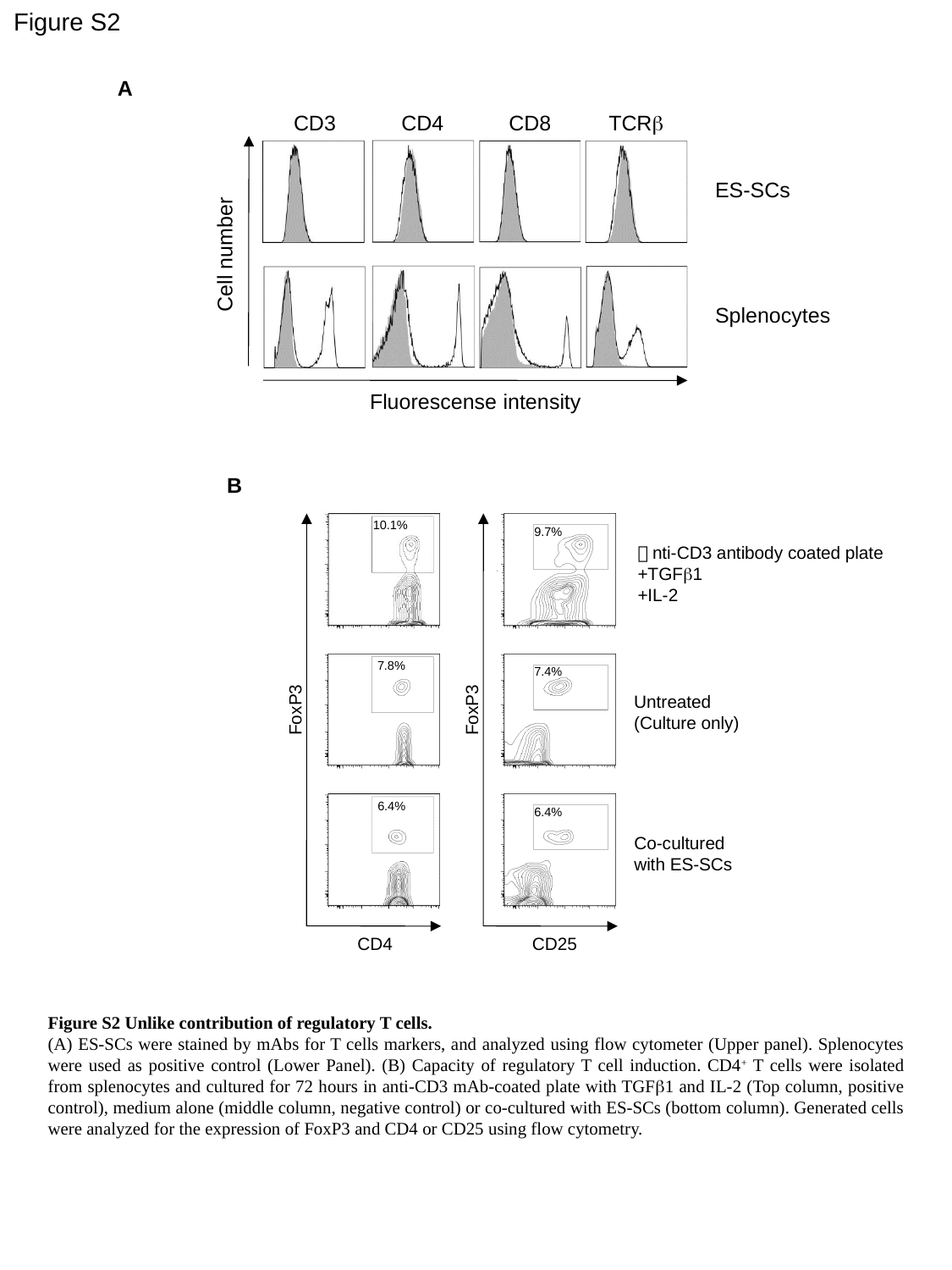

Figure S2
A
CD3
CD4
CD8
TCRβ
ES-SCs
Cell number
Splenocytes
Fluorescense intensity
B
10.1%
FoxP3
CD4
9.7%
Ａnti-CD3 antibody coated plate
+TGFβ1
+IL-2
7.8%
7.4%
Untreated
(Culture only)
FoxP3
6.4%
6.4%
Co-cultured
with ES-SCs
CD25
Figure S2 Unlike contribution of regulatory T cells.
(A) ES-SCs were stained by mAbs for T cells markers, and analyzed using flow cytometer (Upper panel). Splenocytes were used as positive control (Lower Panel). (B) Capacity of regulatory T cell induction. CD4+ T cells were isolated from splenocytes and cultured for 72 hours in anti-CD3 mAb-coated plate with TGFβ1 and IL-2 (Top column, positive control), medium alone (middle column, negative control) or co-cultured with ES-SCs (bottom column). Generated cells were analyzed for the expression of FoxP3 and CD4 or CD25 using flow cytometry.

## Slide 5
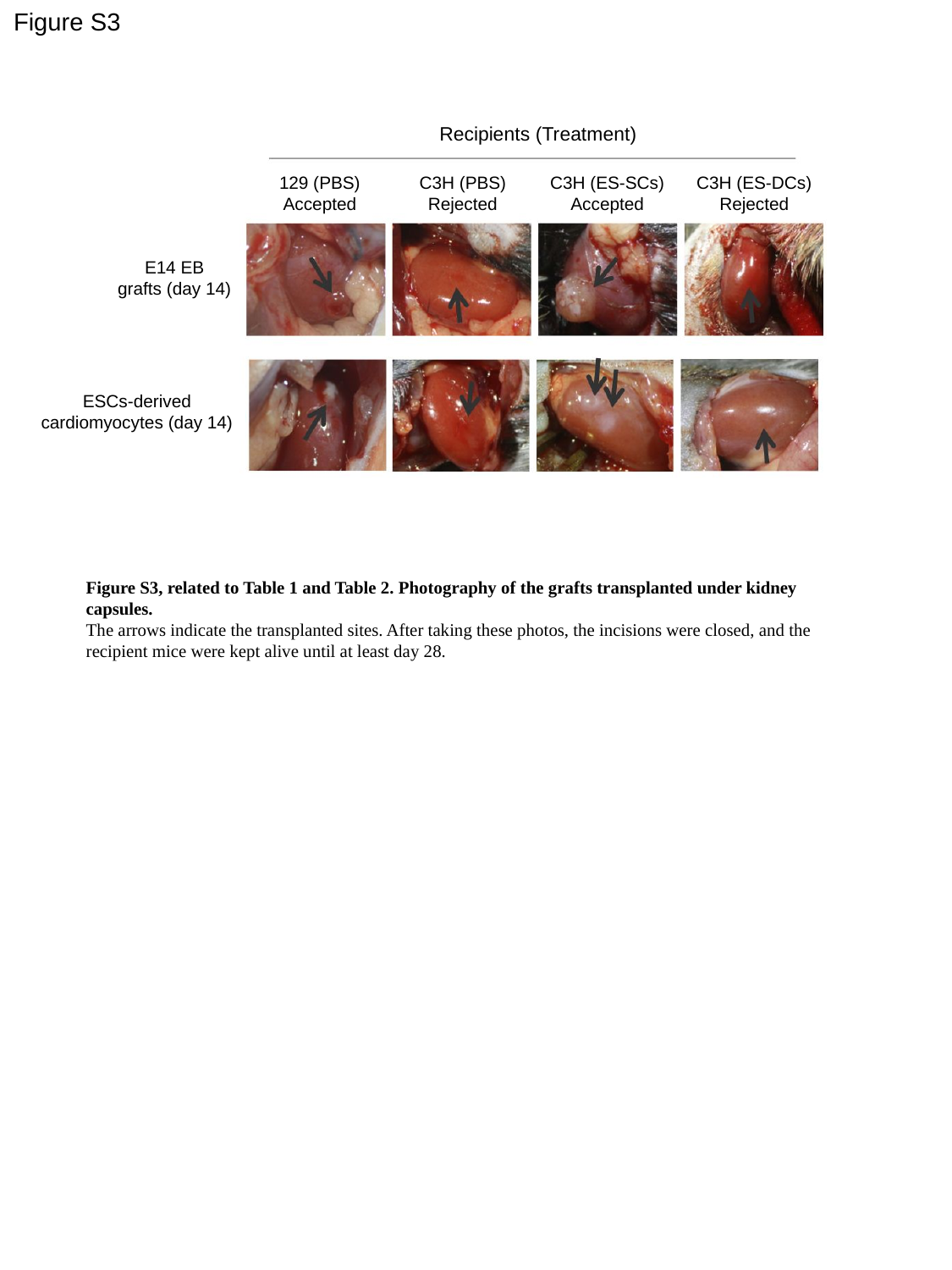

Figure S3
Recipients (Treatment)
129 (PBS)
Accepted
C3H (PBS)
Rejected
C3H (ES-SCs)
Accepted
C3H (ES-DCs)
Rejected
E14 EB
grafts (day 14)
ESCs-derived
cardiomyocytes (day 14)
Figure S3, related to Table 1 and Table 2. Photography of the grafts transplanted under kidney capsules.
The arrows indicate the transplanted sites. After taking these photos, the incisions were closed, and the recipient mice were kept alive until at least day 28.

## Slide 6
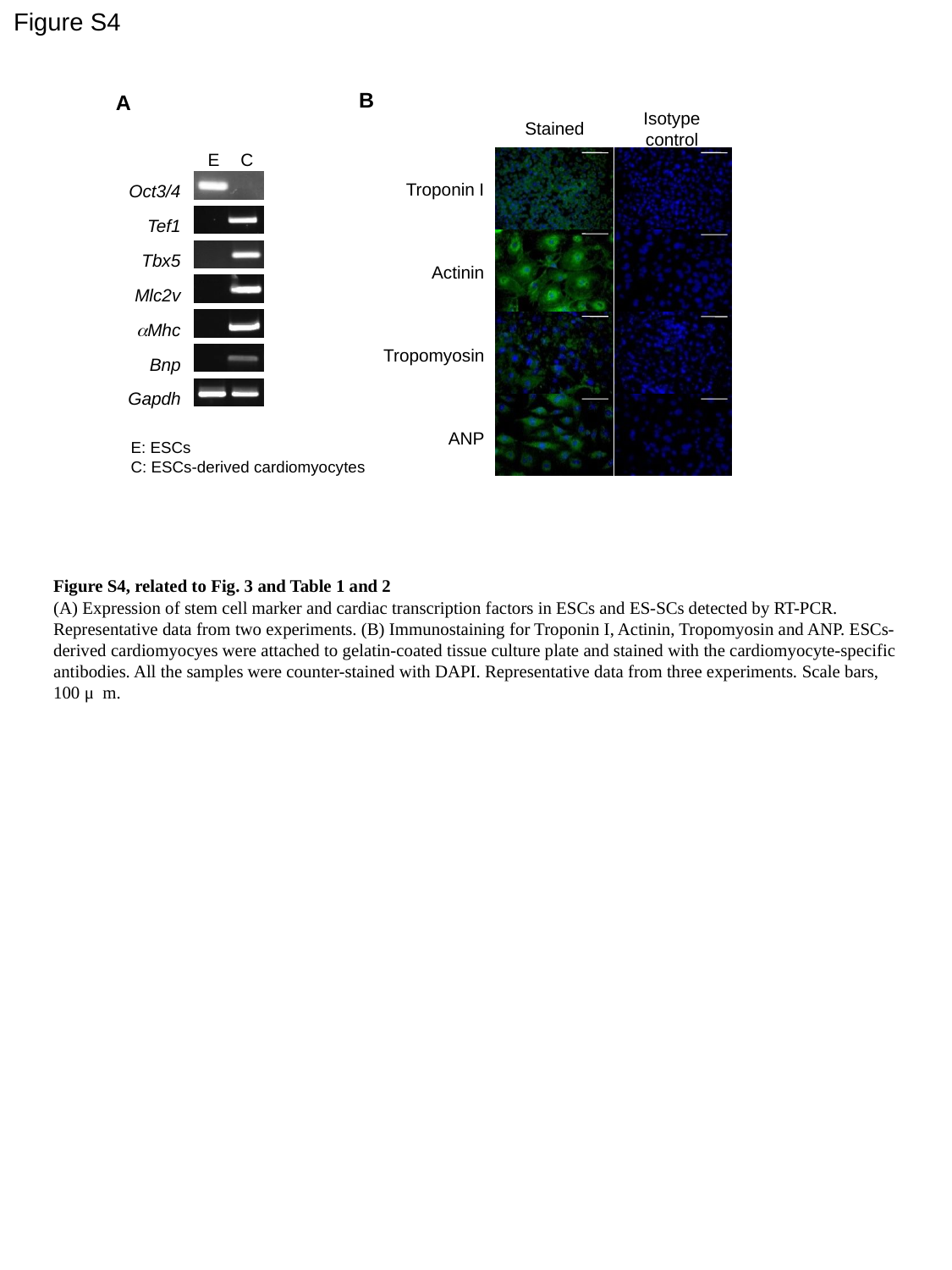

Figure S4
B
A
Isotype control
Stained
E
C
Oct3/4
Troponin I
Tef1
Tbx5
Actinin
Mlc2v
αMhc
Bnp
Tropomyosin
Gapdh
ANP
E: ESCs
C: ESCs-derived cardiomyocytes
Figure S4, related to Fig. 3 and Table 1 and 2
(A) Expression of stem cell marker and cardiac transcription factors in ESCs and ES-SCs detected by RT-PCR. Representative data from two experiments. (B) Immunostaining for Troponin I, Actinin, Tropomyosin and ANP. ESCs-derived cardiomyocyes were attached to gelatin-coated tissue culture plate and stained with the cardiomyocyte-specific antibodies. All the samples were counter-stained with DAPI. Representative data from three experiments. Scale bars, 100 μ m.

## Slide 7
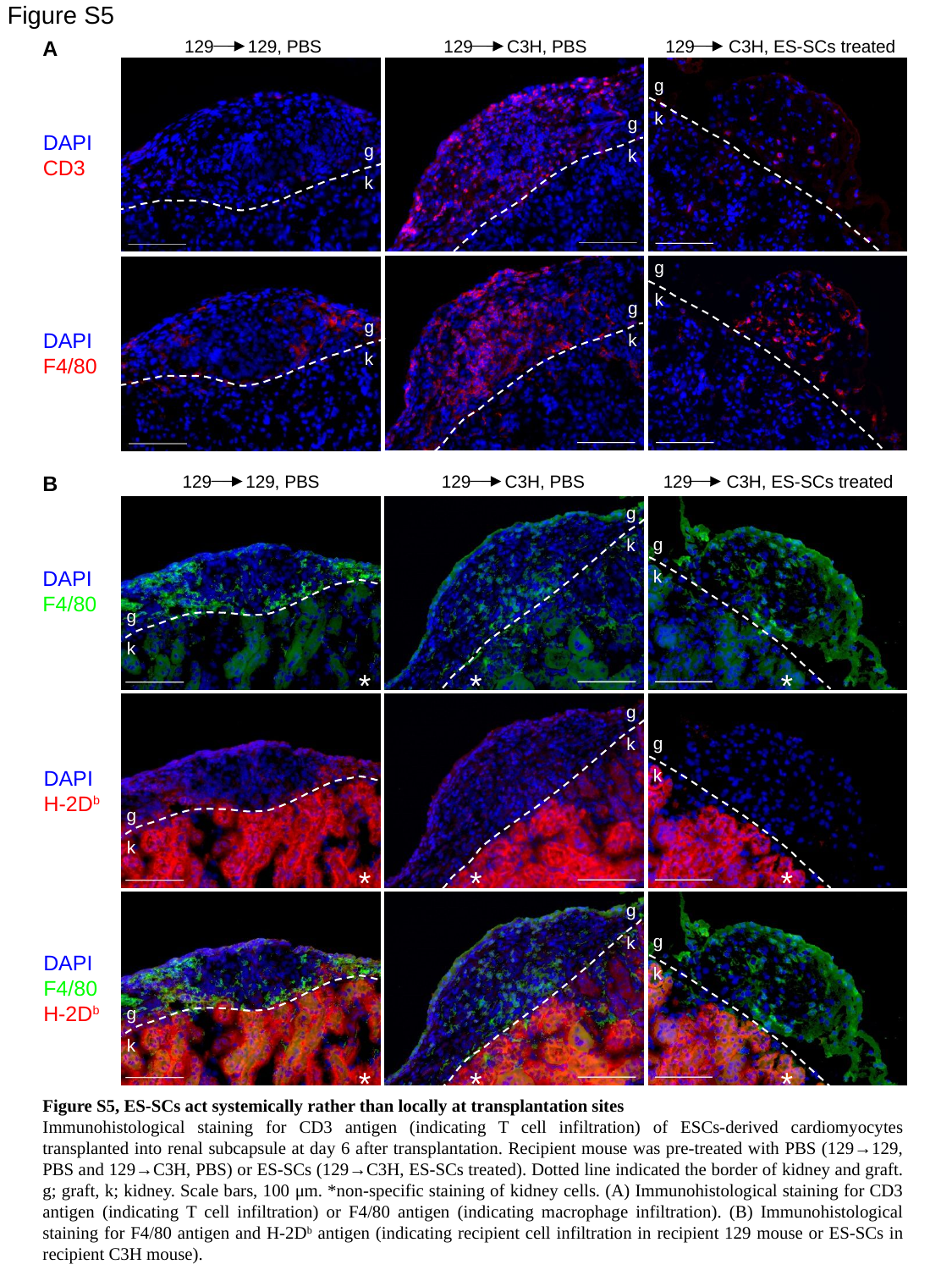

Figure S5
A
129 129, PBS
129 C3H, PBS
129 C3H, ES-SCs treated
g
k
g
DAPI
CD3
g
k
k
g
k
g
g
DAPI
F4/80
k
k
B
129 129, PBS
129 C3H, PBS
129 C3H, ES-SCs treated
g
g
k
k
g
k
DAPI
F4/80
*
*
*
g
g
k
k
g
k
DAPI
H-2Db
*
*
*
g
g
k
k
g
k
DAPI
F4/80
H-2Db
*
*
*
Figure S5, ES-SCs act systemically rather than locally at transplantation sites
Immunohistological staining for CD3 antigen (indicating T cell infiltration) of ESCs-derived cardiomyocytes transplanted into renal subcapsule at day 6 after transplantation. Recipient mouse was pre-treated with PBS (129→129, PBS and 129→C3H, PBS) or ES-SCs (129→C3H, ES-SCs treated). Dotted line indicated the border of kidney and graft. g; graft, k; kidney. Scale bars, 100 μm. *non-specific staining of kidney cells. (A) Immunohistological staining for CD3 antigen (indicating T cell infiltration) or F4/80 antigen (indicating macrophage infiltration). (B) Immunohistological staining for F4/80 antigen and H-2Db antigen (indicating recipient cell infiltration in recipient 129 mouse or ES-SCs in recipient C3H mouse).
