# Supplementary material for: Induction of Macrophage-Like Immunosuppressive Cells from Mouse ES Cells That Contribute to Prolong Allogeneic Graft Survival
Source: PLoS One. 2014 Oct 30;9(10):e111826. doi: 10.1371/journal.pone.0111826 (PMC4214817; doi:10.1371/journal.pone.0111826)
Supplement: Materials and Methods S1 — (PPT) [file pone.0111826.s002.ppt]

## Slide 1
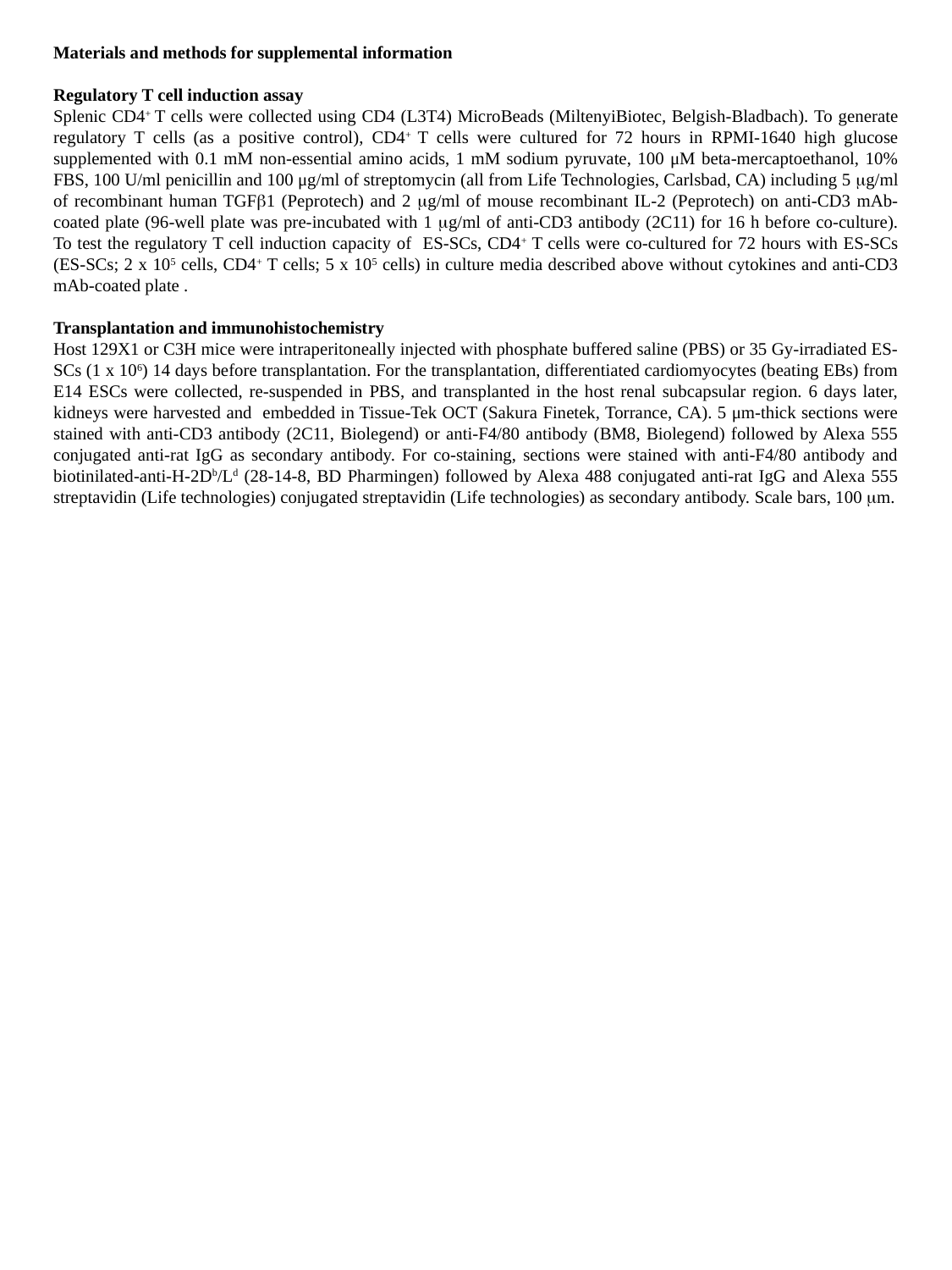

Materials and methods for supplemental information
Regulatory T cell induction assay
Splenic CD4+ T cells were collected using CD4 (L3T4) MicroBeads (MiltenyiBiotec, Belgish-Bladbach). To generate regulatory T cells (as a positive control), CD4+ T cells were cultured for 72 hours in RPMI-1640 high glucose supplemented with 0.1 mM non-essential amino acids, 1 mM sodium pyruvate, 100 μM beta-mercaptoethanol, 10% FBS, 100 U/ml penicillin and 100 μg/ml of streptomycin (all from Life Technologies, Carlsbad, CA) including 5 μg/ml of recombinant human TGFβ1 (Peprotech) and 2 μg/ml of mouse recombinant IL-2 (Peprotech) on anti-CD3 mAb-coated plate (96-well plate was pre-incubated with 1 μg/ml of anti-CD3 antibody (2C11) for 16 h before co-culture). To test the regulatory T cell induction capacity of ES-SCs, CD4+ T cells were co-cultured for 72 hours with ES-SCs (ES-SCs; 2 x 10^5^ cells, CD4+ T cells; 5 x 10^5^ cells) in culture media described above without cytokines and anti-CD3 mAb-coated plate .
Transplantation and immunohistochemistry
Host 129X1 or C3H mice were intraperitoneally injected with phosphate buffered saline (PBS) or 35 Gy-irradiated ES-SCs (1 x 10^6^) 14 days before transplantation. For the transplantation, differentiated cardiomyocytes (beating EBs) from E14 ESCs were collected, re-suspended in PBS, and transplanted in the host renal subcapsular region. 6 days later, kidneys were harvested and embedded in Tissue-Tek OCT (Sakura Finetek, Torrance, CA). 5 μm-thick sections were stained with anti-CD3 antibody (2C11, Biolegend) or anti-F4/80 antibody (BM8, Biolegend) followed by Alexa 555 conjugated anti-rat IgG as secondary antibody. For co-staining, sections were stained with anti-F4/80 antibody and biotinilated-anti-H-2Db/Ld (28-14-8, BD Pharmingen) followed by Alexa 488 conjugated anti-rat IgG and Alexa 555 streptavidin (Life technologies) conjugated streptavidin (Life technologies) as secondary antibody. Scale bars, 100 μm.
